# Reprogramming to Pluripotency Can Conceal Somatic Cell Chromosomal Instability

**DOI:** 10.1371/journal.pgen.1002913

**Published:** 2012-08-30

**Authors:** Masakazu Hamada, Liviu A. Malureanu, Tobias Wijshake, Wei Zhou, Jan M. van Deursen

**Affiliations:** 1 Department of Biochemistry and Molecular Biology, Mayo Clinic, Rochester, Minnesota, United States of America; 2 Department of Pediatric and Adolescent Medicine, Mayo Clinic, Rochester, Minnesota, United States of America; Duke University, United States of America

## Abstract

The discovery that somatic cells are reprogrammable to pluripotency by ectopic expression of a small subset of transcription factors has created great potential for the development of broadly applicable stem-cell-based therapies. One of the concerns regarding the safe use of induced pluripotent stem cells (iPSCs) in therapeutic applications is loss of genomic integrity, a hallmark of various human conditions and diseases, including cancer. Structural chromosome defects such as short telomeres and double-strand breaks are known to limit reprogramming of somatic cells into iPSCs, but whether defects that cause whole-chromosome instability (W-CIN) preclude reprogramming is unknown. Here we demonstrate, using aneuploidy-prone mouse embryonic fibroblasts (MEFs) in which chromosome missegregation is driven by BubR1 or RanBP2 insufficiency, that W-CIN is not a barrier to reprogramming. Unexpectedly, the two W-CIN defects had contrasting effects on iPSC genomic integrity, with BubR1 hypomorphic MEFs almost exclusively yielding aneuploid iPSC clones and RanBP2 hypomorphic MEFs karyotypically normal iPSC clones. Moreover, BubR1-insufficient iPSC clones were karyotypically unstable, whereas RanBP2-insufficient iPSC clones were rather stable. These findings suggest that aneuploid cells can be selected for or against during reprogramming depending on the W-CIN gene defect and present the novel concept that somatic cell W-CIN can be concealed in the pluripotent state. Thus, karyotypic analysis of somatic cells of origin in addition to iPSC lines is necessary for safe application of reprogramming technology.

## Author Summary

iPSC technology has the potential to revolutionize stem-cell based regenerative medicine and would also allow for the production of patient-specific cells for disease modeling and drug discovery. One of the primary safety concerns of iPSCs is genetic instability, which is associated with cancer and various other diseases and includes abnormalities in both chromosomal structure and number. Whereas certain structural chromosome changes have been shown to preclude somatic cell reprogramming, the effect of whole-chromosome reshuffling on this process is completely unknown. Here we show that BubR1 and RanBP2 hypomorphic MEF lines, which are highly prone to erroneous chromosome segregation due to mitotic checkpoint and DNA decatenation failure, respectively, reprogram to pluripotency with normal efficiency. However, while RanBP2 hypomorphic MEFs yielded karyotypically normal iPSC clones with generally low chromosomal instability rates, BubR1 hypomorphic MEFs almost exclusively yielded aneuploid iPSC clones with high instability rates. These data provide important new insights into the genomic integrity requirements during somatic cell reprogramming, and they establish that the safe application of iPSC technology requires screening of both iPSCs and the iPSC-founder cells for chromosome number instability.

## Introduction

The potential to restore pluripotency to mature somatic cells has generated new prospects in the establishment of patient-specific regenerative therapies and has also offered new options for more advanced and specific modeling of human disease [1], [2]. However, several obstacles remain prior to the therapeutic application of iPSCs, including the risk of introducing loss of genomic integrity [3], [4]. Recent studies revealed that somatic cell reprogramming introduces changes at the nucleotide level. Both cell culture length and conditions were identified as key determinants of this type of genetic variation [5], [6]. In contrast to changes at the nucleotide level, reprogramming seems to be less permissive to certain types of structural chromosome damage, such as short telomeres and double strand DNA breaks [7]. Cells with these kinds of aberrations are thought to be eliminated during the early stages of reprogramming by induction of p53-dependent apoptosis [7]. Reprogrammed cells have successfully been generated from somatic cells that undergo stable inheritance of an abnormal number of chromosomes, such as Down syndrome. This implies that aneuploidy (an abnormal number of chromosomes) is not a barrier to reprogramming [8]. However, the extent to which defects that promote the continuous reshuffling of whole chromosomes during mitosis, a condition referred to as whole chromosome instability (W-CIN) [9], interfere with efficient reprogramming of somatic cells is unknown.

The molecular mechanisms that underlie chromosome segregation and that safeguard the process are highly complex and remain incompletely understood [10], [11]. In budding yeast, over one hundred genes are known to cause chromosomal instability when defective, including genes implicated in chromosome condensation, sister chromatid cohesion and decatenation, kinetochore assembly and function, spindle formation, mitotic checkpoint control and attachment error correction [12], [13]. Many more genes are expected to contribute to chromosomal stability in mammals, although only a limited number have been identified to date [9], [14]. To begin to address the impact of numerical chromosome instability, we examined the impact of two distinct W-CIN gene defects on somatic cell reprogramming. The first defect involves the *BubR1* gene, which encodes a core component of the mitotic checkpoint, an intricate surveillance mechanism that acts to delay anaphase onset until all duplicated chromosomes are properly attached to spindle microtubules and aligned in the metaphase plate [15]–[18]. The role of BubR1 in the mitotic checkpoint is to bind to and inhibit Cdc20, a key activator of the anaphase-promoting complex/cyclosome (APC/C) that drives cells into anaphase by targeting cyclin B1 and securin for degradation by the proteasome [19], [20]. In addition, BubR1 functions at kinetochores to stabilize microtubule-chromosome attachments [20], [21]. While complete loss of BubR1 results in cell death by mitotic catastrophe, cells with low amounts of BubR1 are viable despite frequent chromosome missegregation and development of near-diploid aneuploidies [22].

The second defect involves the *RanBP2* gene, which encodes a giant nuclear pore complex (NPC) protein with SUMO E3 ligase activity [23]. At the onset of mitosis, when the nuclear envelope disintegrates and NPCs disassemble, RanBP2 becomes an important regulator of topoisomerase II alpha (hereafter referred to as Top2a), an enzyme that decatenates the centromeric DNA regions of duplicated chromosomes [24]. Accumulation of Top2a at mitotic centromeres is dependent on sumoylation by RanBP2. Complete inactivation of *RanBP2* gene expression results in cell death, but cells with low levels of RanBP2 survive and proliferate normally despite incomplete DNA decatenation, frequent chromosome missegregation and aneuploidization [24].

Here, we show that both W-CIN gene defects are compatible with reprogramming. Unexpectedly, however, the two genetic defects had contrasting effects on the genomic integrity of the reprogrammed cells, with RanBP2-insufficient MEFs generating karyotypically normal and chromosomally stable iPSCs and BubR1-insufficient MEFs almost exclusively yielding aneuploid and chromosomally unstable iPSC clones. These data indicate that aneuploid cells can be selected for or against during reprogramming depending on the genetic defect driving the chromosome number instability. Furthermore, our data reveal that W-CIN that exists at the somatic cell level can become dormant upon reprogramming, indicating that testing of both iPSCs and the iPSC-founding cells for chromosome number instability will be necessary for the safe application of iPSC technology in regenerative medicine.

## Results

### W-CIN Is Not a Barrier for Cellular Reprogramming

**Figure 1:**
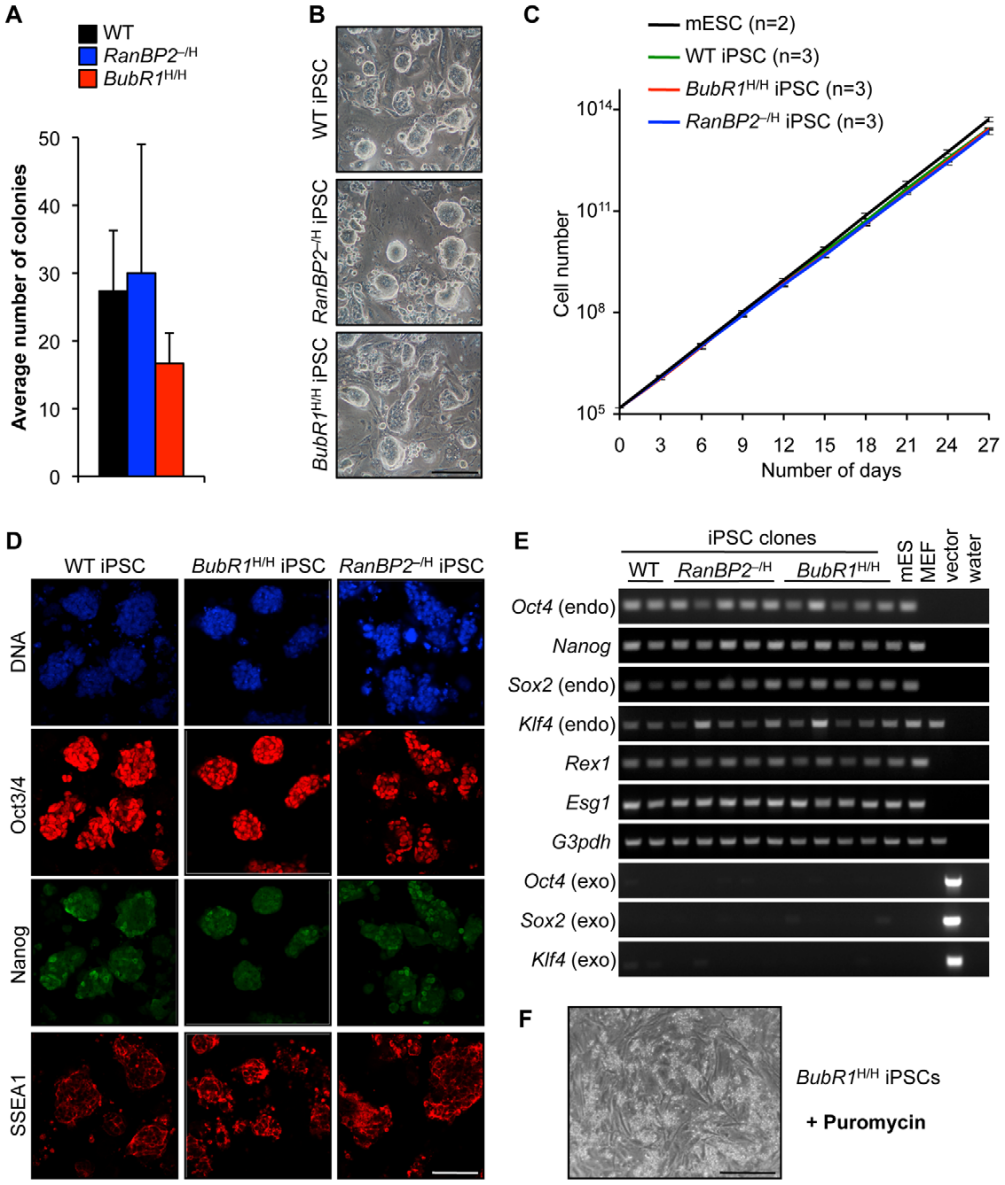
MEFs with W-CIN gene mutations can be efficiently reprogrammed into iPSCs. (A) Average numbers of ES cell-like colonies derived from MEFs with indicated genotypes (n = 3 independent MEF lines). Error bars represent SEM. (B) Light microscopy images of iPSC colonies with indicated genotypes growing on SNL feeder layer. Scale bar represents 500 µm. (C) Growth curves of mESCs and iPSC clones derived from MEFs of the indicated genotypes. We note that there were no significant differences in growth between individual iPSC lines of each genotype. Error bars represent SD. (D) Immunostaining of wildtype, *RanBP2* ^–/H^ and *BubR1* ^H/H^ iPSC for ES cell markers. Representative images are shown. DNA of cells stained for Oct3/4 and Nanog was visualized with Hoechst. Scale bar represents 200 µm. (E) RT-PCR analysis of retroviral transgene silencing and endogenous pluripotency-associated gene induction. (F) Silencing of retroviral expression after direct reprogramming. *BubR1* ^H/H^ iPSCs die upon addition of 4 µg/ml puromycin for 24 h. Scale bar represents 500 µm.

We investigated the impact of W-CIN on cell reprogramming using MEFs derived from *BubR1* (*BubR1* ^H/H^) and *RanBP2* hypomorphic (*RanBP2* ^–/H^) mutant mice [22], [24]. Earlier work demonstrated that *BubR1* ^H/H^ MEFs generate ∼10% of normal BubR1 protein levels and *RanBP2* ^–/H^ mice ∼26% of normal RanBP2 protein levels. We selected *BubR1* ^H/H^ and *RanBP2* ^–/H^ MEFs for our studies because their aneuploidy rates are quite similar, with *BubR1* ^H/H^ cultures having 36% aneuploid cells at passage 5 (P5) [25] and *RanBP2* ^–/H^ cultures 33% [24]. Moreover, entirely different mechanisms drive aneuploidization in *BubR1* ^H/H^ and *RanBP2* ^–/H^ MEFs. Newly performed chromosome counts on P5 wildtype, *BubR1* ^H/H^ and *RanBP2* ^–/H^ MEFs confirmed our previously published aneuploidy rates for these genotypes (Table 1). To induce reprogramming to pluripotency, Oct-3/4, Sox2, and Klf4 expression constructs were introduced in P5 wildtype, *BubR1* ^H/H^, and *RanBP2* ^–/H^ MEFs by retroviral transduction. c-Myc was omitted because its overexpression has been associated with aneuploidization [26]. Embryonic stem (ES) cell-like colonies emerged around two weeks after transduction, irrespective of genotype. The number of ES cell-like colonies emerging from *BubR1* ^H/H^ or *RanBP2* ^–/H^ MEF lines were similar to those originating from wildtype MEFs (Figure 1A). The finding that ES-like colonies developed from *BubR1* ^H/H^ MEFs with normal efficiency was somewhat unexpected because p19^Arf^, p16^Ink4a^ and p53 are elevated in P5 *BubR1* ^H/H^ MEFs [25], [27] and have previously been shown to impair reprogramming [7], [28]. We found that growth rates of P5 *BubR1* ^H/H^ MEFs were similar to those of P5 wildtype and *RanBP2* ^–/H^ MEFs (Figure S1), implying that the level of p19^Arf^, p16^Ink4a^ and p53 engagement was too low to have a substantial impact on global cell cycle progression.

**Table 1:** MEF aneuploidy rates prior to reprogramming.

MEFgenotype	Line	Mitoticcellsinspected	Percentaneuploidcells	Karyotypes with the indicated chromosome number									
				35	36	37	38	39	40	41	42	43	44
**Wildtype**	1	50	12	1	1		1	1	44	2			
	2	50	10				1	2	45	2			
	3	50	8					1	46	2		1	
***BubR1*** **^H/H^**	1	50	38	1		3	1	5	31	1	4	3	1
	2	50	40	3		2	3	3	30	3	4	1	1
	3	50	36		4	3	1	2	32	4	2	2	
***RanBP2*** **^–/H^**	1	50	30		2		2	1	35	6	1	3	
	2	50	34			2	2	7	33	5	1		
	3	50	34		2	1	2	5	33	2	3	2	

Chromosome counts were performed at P5.

Individual ES cell-like colonies were picked and clonally expanded on monolayers of mitotically inactivated STO feeder cells that exogenously express NEO and leukemia inhibitory factor (LIF), referred to as SNL feeders [29]. The morphology and growth rate of these cells was similar to that of murine ES cells (ESCs) derived from 129Sv/E×C57BL/6 blastocysts, irrespective of genotype (Figure 1B and 1C). We used immunofluorescence to screen for the presence of ES cell-associated markers. Reprogrammed cells derived from all three MEF genotypes consistently expressed Oct3/4, Nanog, and SSEA1, with each marker exhibiting the proper fluorescence intensity and subcellular localization (Figure 1D). RT-PCR analysis confirmed that *Oct4* and *Nanog* expression was elevated, together with several other ES cell-associated marker genes (Figure 1E). Consistent with reprogramming [30], [31], retroviral expression of Yamanaka factors was silenced in iPSCs of all three genotypes (Figure 1E). Furthermore, the puromycin resistance gene, which is co-expressed with exogenous *Oct-3/4*, *Sox2*, and *Klf4* from an IRES [1], was co-silenced upon reprogramming as evidenced by prompt iPSC death in the presence of puromycin (Figure 1F).

**Figure 2:**
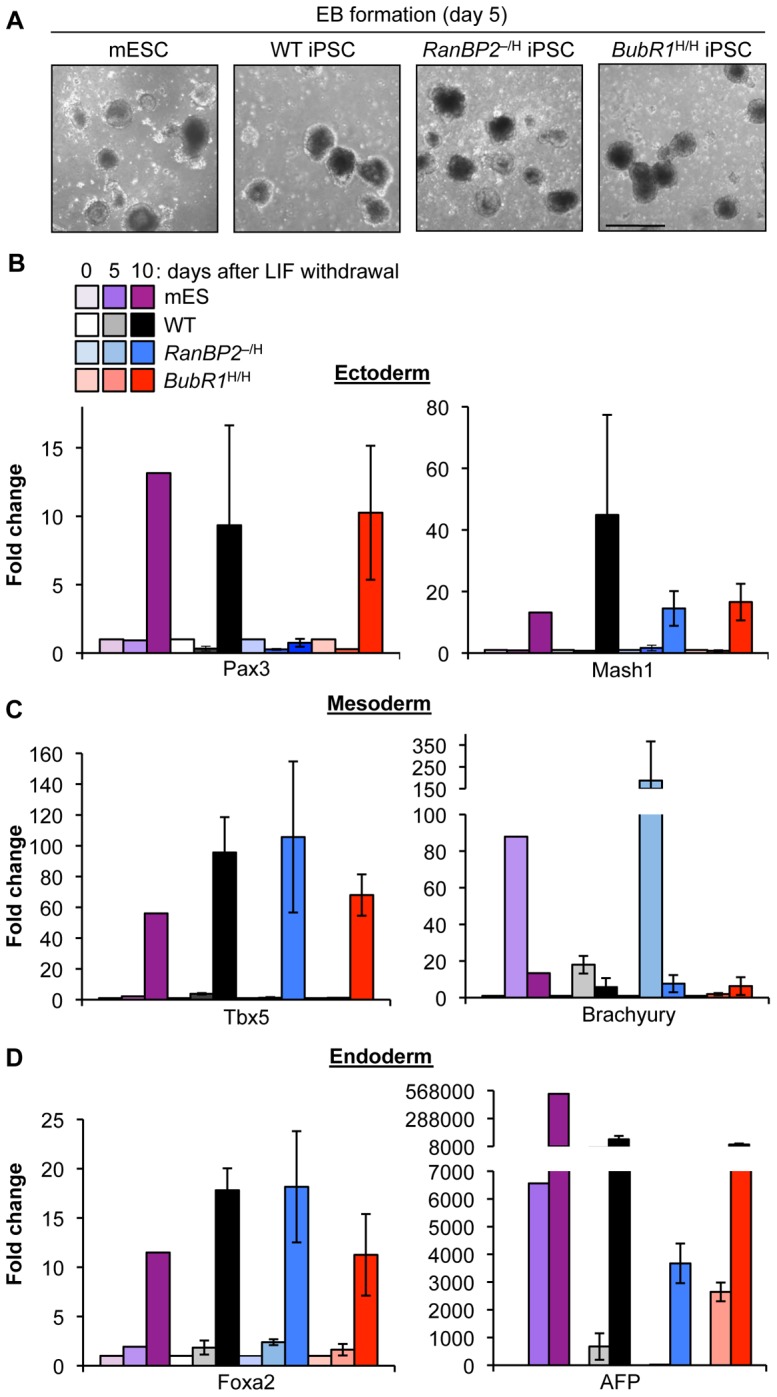
iPSCs from W-CIN mutant MEFs differentiate into EBs comprising of all embryonic germ layers. (A) Representative EBs derived from mESCs and iPSC clones. (B–D) qRT-PCR analysis of EBs derived from mESCs and iPSC clones for expression of the indicated embryonic germ layer markers. Error bars represent SEM.

**Figure 3:**
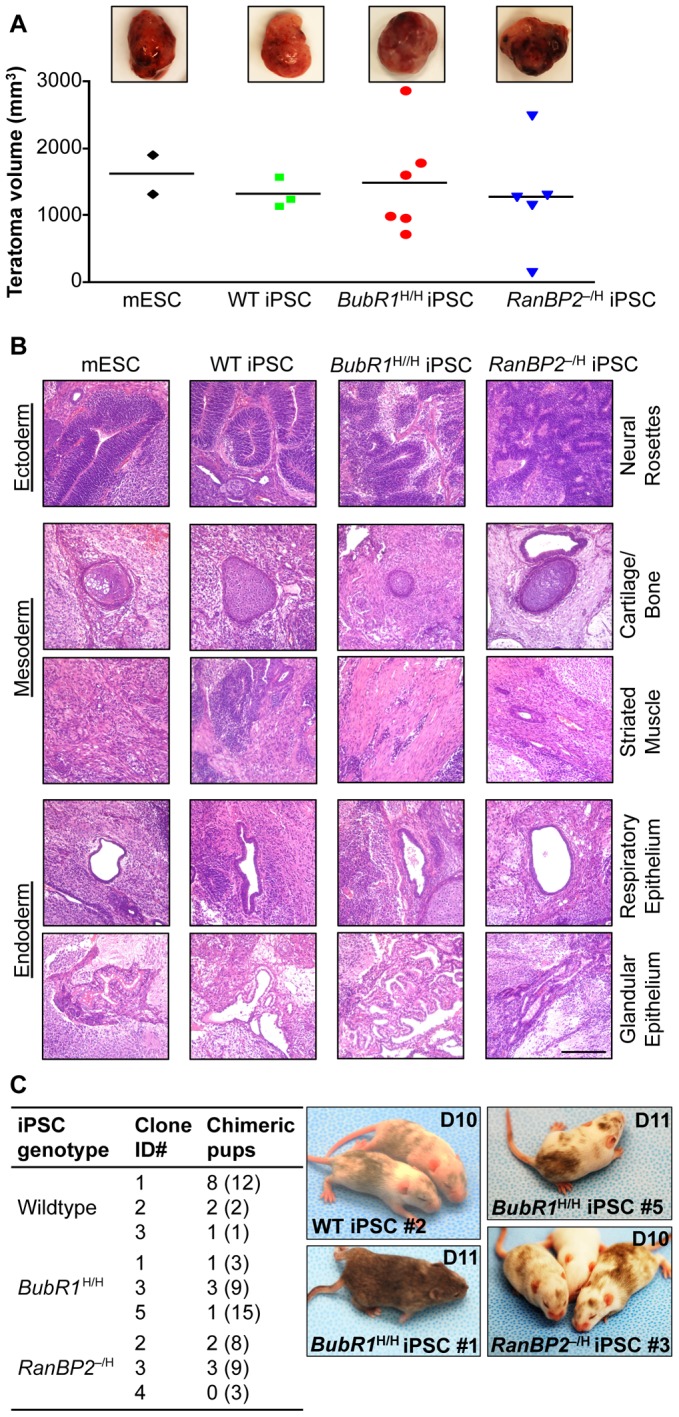
iPSCs derived from W-CIN MEFs form teratomas and chimeric mice. (A) Analysis of teratomas derived from iPSC clones of the indicated genotypes. mESCs were used as a control for teratoma formation. (Top) Images of representative teratomas collected 21 days after injection of iPSCs into SCID mice. (Bottom) Teratoma volume plotted as a scatter plot with mean. We note that *RanBP2* ^–/H^ and *BubR1* ^H/H^ MEFs failed to form teratomas. (B) Hematoxylin and eosin staining of teratoma sections. Scale bar is 200 µm. (C) Aneuploid iPSCs injected into BALB/c host blastocysts produce viable chimeric mice. (Left) Summary of blastocyst injection results. Clone ID#s correspond to iPSC clones listed in Table 2. Numbers in parenthesis indicate total number of pups delivered. (Right) Images of chimeric animals produced by blastocyst injection of iPSCs. The ages of the pups are indicated in days.

To further evaluate whether *BubR1* ^H/H^ and *RanBP2* ^–/H^ iPSC clones were properly reprogrammed, we tested their developmental potential by several methods. First, we formed embryoid bodies (EBs) by growing wildtype, *BubR1* ^H/H^ and *RanBP2* ^–/H^ iPSC clones in suspension in ES medium lacking LIF (Figure 2A). EBs harvested at day 5 and 10 expressed ectodermal (*Pax3* and *Mash1*), mesodermal (*Tbx5* and *Brachyury*), and endodermal (*AFP* and *Foxa2*) markers irrespective of genotype [32] (Figure 2B–2D), indicating that all three germ layers were present. Second, when injected subcutaneously into SCID mice, wildtype (n = 3), *BubR1* ^H/H^ (n = 6) and *RanBP2* ^–/H^ iPSC clones (n = 5) produced aggressively growing teratomas that contained tissue structures representing all three embryonic germ layers (Figure 3A and 3B). Third, when injected into blastocysts, wildtype (n = 3), *BubR1* ^H/H^ (n = 3), and *RanBP2* ^–/H^ iPSC clones (n = 3) produced viable chimeric animals, with the exception of one *RanBP2* ^–/H^ iPSC clone (Figure 3C). Failure to generate chimeras by this *RanBP2* ^–/H^ iPSC clone was most likely due to the low number of pups born rather than lack of pluripotency as it was capable of forming teratomas in which cell types derived from the three germ layers were detectable. Collectively, the above data suggest that MEFs with W-CIN gene defects fully reprogram to iPSCs with similar efficiency as wildtype MEFs.

### Reprogramming Ability of Aneuploid Cells Is W-CIN Gene–Dependent

Next, we performed chromosome counts on metaphase spreads of independent *RanBP2* ^–/H^ and *BubR1* ^H/H^ iPSC clones to determine whether there might be a bias against reprogramming of aneuploid MEF cells and to compare W-CIN rates before and after reprogramming. As shown in Table 2, all ten *RanBP2* ^–/H^ iPSC clones examined predominantly consisted of cells with 40 chromosomes, indicating that they originated from karyotypically normal MEF cells. In contrast, only one of 11 *BubR1* ^H/H^ iPSC clones analyzed predominantly consisted of cells with 40 chromosomes, implying that 10 clones originated from aneuploid *BubR1* ^H/H^ MEFs (Table 2). However, an alternative explanation for the data would be that reprogramming is restricted to karyotypically normal *BubR1* ^H/H^ MEFs but that massive aneuploidization initiated after the completion of reprogramming accounts for the genesis of iPSC clones where the predominant population of cells has a chromosome number other than 40 (see Figure S2). To explore the probability of this model, we selected three independent *BubR1* ^H/H^ iPSC clones listed in Table 2 (each having a majority population of cells with a different chromosome number), prepared subclones from single cells and analyzed their karyotypes. As shown in Table 3, in each instance, the majority of the subclones maintained a karyotype that closely resembled that of the parental line. Specifically, in most subclones the majority of the cells had a chromosome number found in the majority cells of the corresponding parental clone. These data argue against the notion that severe aneuploidization at the early stages of clonal expansion of reprogrammed cells accounts for prevalence of aneuploid *BubR1* ^H/H^ iPSC clones, and are consistent with the idea that aneuploid *BubR1* ^H/H^ MEFs reprogram more efficiently than *BubR1* ^H/H^ MEFs with a normal karyotype. Wildtype MEFs, which typically have aneuploidy rates of ∼9% at P5 [22], [24], showed a moderate bias for reprogramming of aneuploid MEFs, with 23% of iPSC clones analyzed originating from aneuploid MEF cells. Thus, perhaps biased reprogramming of karyotypically abnormal MEFs may not be restricted to *BubR1* ^H/H^ cells.

**Table 2:** W-CIN gene defects do not preclude somatic cell reprogramming.

iPSCgenotype	iPSCcloneID#	Mitoticcellsinspected	Percentaneuploidcells	Percentdeviation	Karyotypes with the indicated chromosome number										
					35	36	37	38	39	40	41	42	43	44	45
***RanBP2*** **^–/H^**	1	50	4	4			1		1	**48**					
	2	50	6	6				2		**47**	1				
	3	50	8	8			1		2	**46**	1				
	4	50	8	8			1		1	**46**	1	1			
	5	50	10	10					2	**45**	3				
	6	50	10	10					4	**45**	1				
	7	50	12	12			1	1	2	**44**	2				
	8	50	16	16			2	1	2	**42**	2	1			
	9	50	18	18			1		5	**41**	3				
	10	50	28	28		1	3	1	7	**36**	2				
***BubR1*** **^H/H^**	1	100	53	53	1	3	2	7	19	**47**	20	1			
	2	50	76	42			1	1		12	**29**	6	1		
	3	50	90	48				1		5	**26**	16	2		
	4	50	78	76		1		2	2	11	**12**	8	10	3	1
	5	50	92	46		1	1	1	1	4	3	**27**	11	1	
	6	50	96	58	1		1	2		2	17	**21**	5	1	
	7	50	82	66			4	1	1	9	13	**17**	5		
	8	50	94	60						3	9	**20**	11	5	2
	9	50	98	60		1	2			1	4	15	**20**	3	4
	10	50	96	68		1		2	1	2	8	12	**16**	7	1
	11	50	98	48		1	2	1		1	4	4	8	**26**	3
**Wildtype**	1	50	12	12			1	1	2	**44**		1	1		
	2	50	12	12				1	3	**44**		2			
	3	50	14	14			1		3	**43**	3				
	4	50	16	16			1	1	4	**42**	1		1		
	5	50	18	18				1	1	**41**	7				
	6	50	20	20			2	2	5	**40**	1				
	7	50	20	20		1		2	6	**40**	1				
	8	50	28	28	1		1	4	4	**36**	4				
	9	50	34	34					1	**33**	15	1			
	10	50	36	36	1	1	1	2	7	**32**	3		1		2
	11	50	90	16				2	**42**	5	1				
	12	50	92	18	1		1	1		4	**41**	2			
	13	50	98	16					1	1	3	**42**	2	1	

Chromosome counts on iPSC clones with indicated genotypes. Percent deviation indicates the fraction of spreads with the chromosome number different from the most frequent count.

**Table 3:** Karyotypes of subcloned BubR1 hypomorphic iPSCs reflect the parental kayotype profile.

Parental*BubR1* ^H/H^iPSC clone	iPSCID#	Mitoticcellsinspected	Percentaneuploidcells	Percentdeviation	Karyotypes with the indicated chromosome number										
					35	36	37	38	39	40	41	42	43	44	45
**Clone 1**	*Parental*	*100*	*53*	*53*	*1*	*3*	*2*	*7*	*19*	***47***	*20*	*1*			
	Subclone 1	50	46	46		1	1	2	9	**27**	7	3			
	Subclone 2	50	44	44	2	1	1	4	6	**28**	6	1	1		
	Subclone 3	50	44	44			2	1		**28**	17	2			
	Subclone 4	50	40	40			5	5	5	**30**	4	1			
	Subclone 5	50	40	40		1		2	10	**30**	7				
	Subclone 6	50	34	34		2		2	8	**33**	3	2			
	Subclone 7	50	20	20	2				6	**40**	2				
	Subclone 8	50	88	46	3	2	1	2	3	6	**27**	6			
	Subclone 9	50	90	40	1	1				5	**30**	8	5		
	Subclone 10	50	80	32	1	1		2		10	**34**	2			
**Clone 5**	*Parental*	*50*	*92*	*46*		*1*	*1*	*1*	*1*	*4*	*3*	***27***	*11*	*1*	
	Subclone 1	50	84	64					4	8	17	**18**	3		
	Subclone 2	50	100	46							11	**27**	12		
	Subclone 3	50	96	46					1	2	14	**27**	6		
	Subclone 4	50	96	42	1		1		1	2	5	**29**	9	2	
	Subclone 5	50	92	44				1		4	9	**28**	8		
	Subclone 6	50	96	40		1		2		2	8	**30**	6	1	
	Subclone 7	50	94	56		2		1	1	3	4	8	**22**	6	3
	Subclone 8	50	98	52					1	1	2	9	**24**	13	
	Subclone 9	50	100	50				1			12	12	**25**		
	Subclone 10	50	100	36			1					14	**32**	3	
**Clone 11**	*Parental*	*50*	*98*	*48*		*1*	*2*	*1*		*1*	*4*	*4*	*8*	***26***	*3*
	Subclone 1	50	90	58					1	5	2	3	15	**21**	3
	Subclone 2	50	96	46			1	1		2	5	3	11	**27**	
	Subclone 3	50	96	44			1	1		2	3	5	8	**28**	2
	Subclone 4	50	98	50		1	2		2	1	5	3	6	**25**	5
	Subclone 5	50	94	56	1		1		1	3	5	8	3	**22**	6
	Subclone 6	50	98	46	1			1	1	1	1	6	**27**	11	1
	Subclone 7	50	98	52			1	2	1	1	5	**24**	14	1	1
	Subclone 8	50	100	34				2	2		8	**33**	4	1	
	Subclone 9	50	96	32				1	1	2	8	**34**	2	2	
	Subclone 10	50	94	56		1		1		3	1	1	5	16	**22**

Parental IPSC clone ID#s correspond to clones listed in Table 2. The most frequent count of each iPSC subclone is in bold typeface. Percent deviation indicates the fraction of spreads with the chromosome number different from the most frequent count.

**Figure 4:**
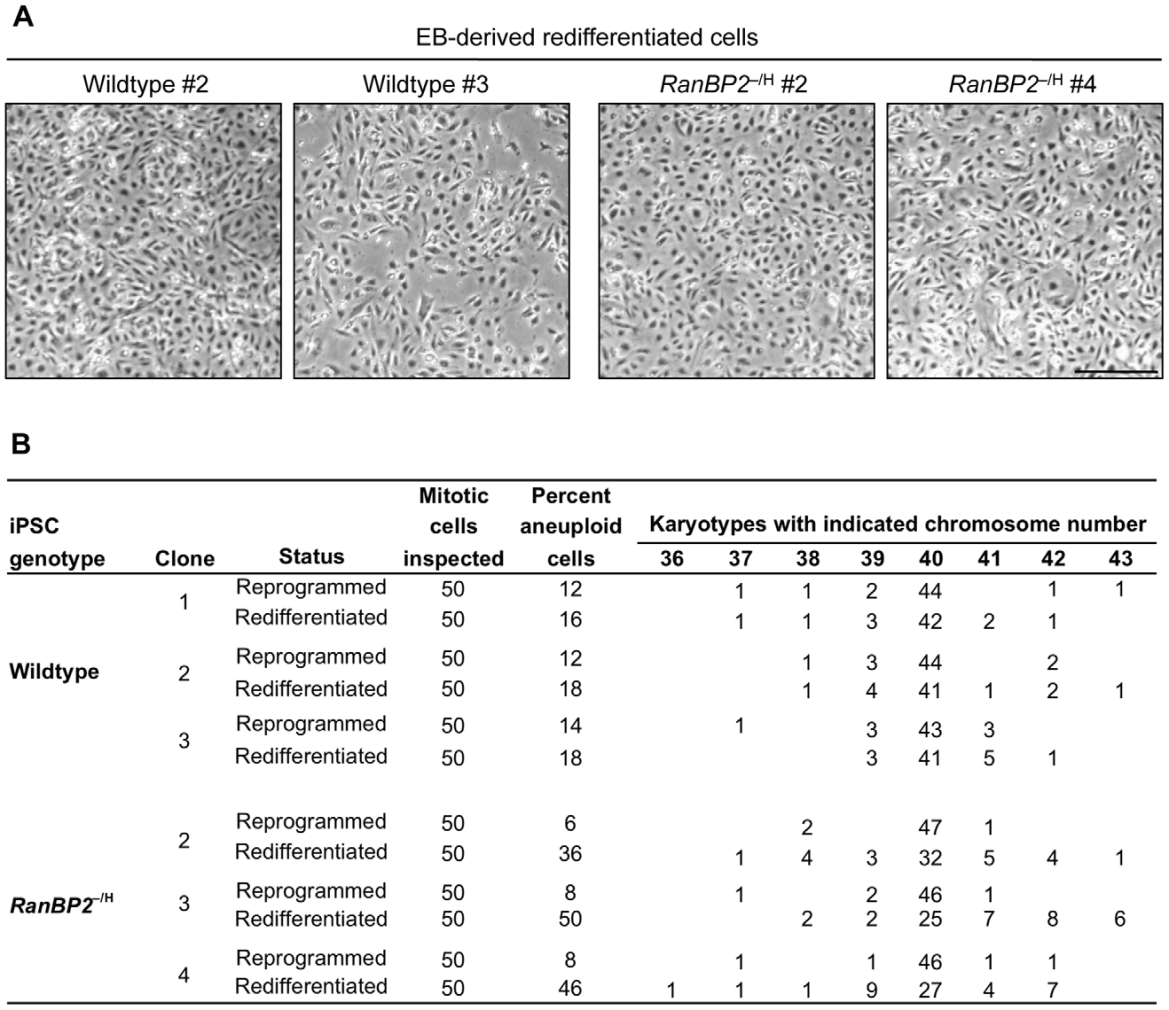
RanBP2 hypomorphic iPSCs reestablish W-CIN after redifferentiation. (A) Light microscopy images of cell cultures derived from EBs with indicated genotypes. Scale bar represents 500 µm. (B) Chromosome counts on EB-derived cells paired with the karyotype of the parental iPSC clone from Table 2.

*RanBP2* ^–/H^ iPSC clones on average had a much lower percentage of aneuploid cells (12%±7%; Table 2) than *RanBP2* ^–/H^ MEFs (33%±2%; Table 1). This reveals that RanBP2 insufficiency has a differential impact on the accuracy of chromosome segregation in pluripotent and differentiated cells, indicating that certain W-CIN gene defects can be masked during reprogramming. Redifferentiation of *RanBP2* ^–/H^ iPSC clones with low rates of aneuploidy resulted in a dramatic increase in aneuploidization (Figure 4A and 4B). In contrast, redifferentiation of wildtype iPSC clones resulted only in very modest increases in aneuploidy. These data argue against the possibility that reprogramming of *RanBP2* ^–/H^ MEFs select for compensatory genetic alterations that improve chromosome segregation fidelity and further support the notion that chromosomal instability associated with RanBP2 hypomorphism is masked at the pluripotent state.

### Reprogramming Reduces Dependence of Sister Centromere Decatenation on RanBP2

**Figure 5:**
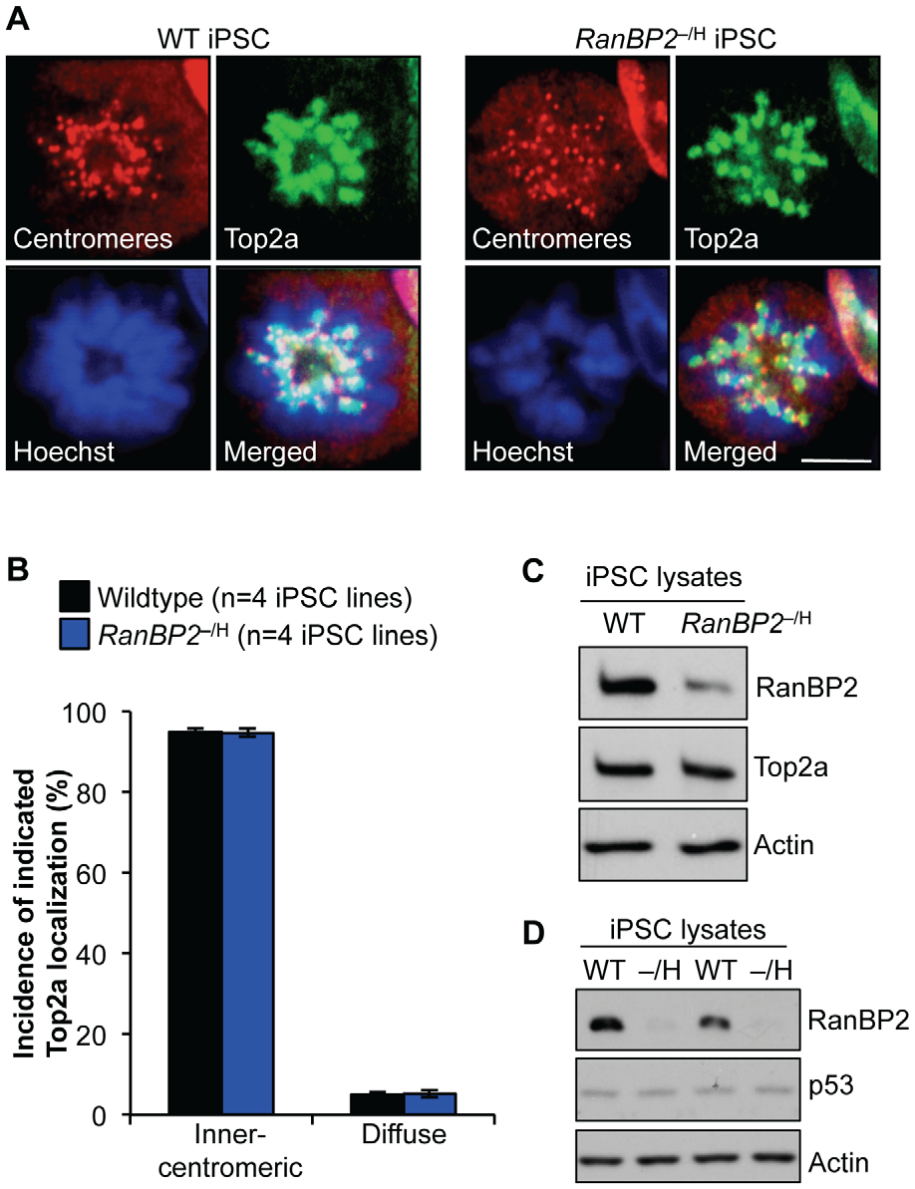
*RanBP2* ^–/H^ iPSCs efficiently recruit Top2a to the inner centromeres. (A) Immunolocalization of Top2a in prometaphase of wildtype and *RanBP2* ^–/H^ iPSCs. Centromeres are visualized with ACA antibody. DNA was stained with Hoechst. Scale bar represents 10 µm (2000× magnification) (B) Quantification of prometaphases with inner centromeric versus diffuse localization of Top2a. At least 50 prometaphases were analyzed per genotype. Error bars represent SD. (C) Western blot analysis of iPSC extracts probed for RanBP2 and Top2a. Actin served as a loading control. (D) Western blot analysis of iPSC extracts probed for RanBP2 and p53. Actin served as a loading control.

Decatenation of centromeric DNA by Top2a is essential for proper chromosome separation of sister chromatids [33]. Previously, we showed that targeting of Top2a to inner centromeres of mitotic chromosomes is regulated by RanBP2-mediated sumoylation [24]. Based on these earlier findings, we proposed that the marked decrease in aneuploidization upon reprogramming of *RanBP2* ^–/H^ MEFs might be due to improved centromeric targeting of Top2a. To explore this hypothesis, iPSC clones derived from wildtype and *RanBP2* ^–/H^ MEFs were stained with antibodies against Top2a and centromeres. As expected, nearly all wildtype iPSCs accumulated Top2a to the inner centromeres at mitosis onset (Figure 5A and 5B). Consistent with correction of W-CIN upon reprogramming, *RanBP2* ^–/H^ iPSCs localized Top2a to the inner centromeres with similar efficiency as iPSC derived from wildtype MEFs [24].

It has been reported that a significant number of proteins implicated in cell cycle regulation are upregulated in pluripotent stem cells, including RanBP2 [34], which led us to speculate that normalization of Top2a localization in *RanBP2* ^–/H^ iPSC cells might be due to loss of RanBP2 hypomorphism during reprogramming. To test this, we used western blot analysis to compare RanBP2 protein levels in iPSCs derived from wildtype and *RanBP2* ^–/H^ iPSC clones. We observed that RanBP2 levels were indeed reduced in *RanBP2* ^–/H^ iPSCs (Figure 5C and Figure S3) and that the level of reduction was similar to that documented for *RanBP2* ^–/H^ MEFs [24]. Furthermore, Top2a levels were normal in *RanBP2* ^–/H^ iPSCs, indicating that the mechanism of correction of Top2a localization to inner centromeres does not involve compensatory expression (Figure 5C). Furthermore, it has been proposed that p53 safeguards against aneuploidy [35], [36], which led us to speculate that RanBP2 insufficiency might sensitize reprogrammed cells to aneuploidy-induced p53 induction, thereby allowing for more efficient elimination of aneuploid iPSCs from *RanBP2* ^–/H^ iPSC cultures. We explored this potential mechanism by measuring p53 levels in extracts prepared from *RanBP2* ^–/H^ and WT iPSC cultures, but no detectable differences in p53 levels were observed between the two genotypes (Figure 5D). Taken together, the above data indicate that pluripotent cells are less dependent on RanBP2 for proper Top2a targeting to inner centromeres than somatic cells.

### Subcloning of iPSCs Can Improve Chromosome Number Integrity

Our finding that cultures of wildtype iPSCs originating from karyotypically normal MEF cells have aneuploidy rates of 12–36% highlights that reprogramming is subject to substantial cell culture induced aneuploidy (Table 2). High rates of chromosomal instability pose a potential safety risk in regenerative therapies based on iPSCs, since aneuploidization is potentially tumor promoting [9], [10], [37]. Furthermore, because aneuploidy can alter the metabolic and proliferative properties of cells [4], [38], [39], aneuploidization of cultured iPSCs may impact the analysis of studies using disease-specific iPSCs derived from patients. It is therefore important to identify methods for selection of pluripotent cells with normal karyotypes and for maintenance of karyotypic stability during cell culture. To test if aneuploidy rates of wildtype iPSC clones can be decreased through subcloning, we generated subclones from three independent wildtype iPSC cultures (clone ID# 1, 2 and 3 in Table 2), which had aneuploidy rates of 12%, 12% and 14%, respectively. Subclones of each of these iPSC clones were expanded and subjected to chromosome counts (at P3 after picking). Although none of the subclones analyzed consisted of cells with only 40 chromosomes (Table 4), 8 out of 19 subclones had at least two fold reduced aneuploidy rates compared to their parental iPSC clones, with 2 subclones containing 2% aneuploidy and four subclones containing 4% aneuploidy. Only three of the subclones had substantially higher aneuploidy rates. When re-examined after 6 additional passages, 3 of 4 subclones with an improved karyotype showed persistence of the upgrade (Table 4). Thus, although subcloning could not entirely eliminate aneuploidy, it yielded several iPSC cultures with continued low rates of karyotypic instability.

**Table 4:** Subcloning can reduce aneuploidy rates of iPSC clones.

ParentaliPS clone	iPSCID#	Mitoticcellsinspected	Percentaneuploidcells	Karyotypes with the indicated chromosome number								
				36	37	38	39	40	41	42	43	44
**Wildtype 1**	*Parental*	*50*	*12*		*1*	*1*	*2*	*44*		*1*	*1*	
	*Subclone 1*	*50*	*6*				*3*	*47*				
	Subclone 2	50	10				1	45	4			
	Subclone 3	50	10				3	45	1	1		
	Subclone 4	50	14			1		43	4	1	1	
	Subclone 5	50	28	2	1	1	2	36	6		1	1
**Wildtype 2**	*Parental*	*50*	*12*			*1*	*3*	*44*		*2*		
	*Subclone 1*	*50*	*2 (4)*				*(2)*	*49 (48)*	*1*			
	*Subclone 2*	*50*	*2 (4)*				*(1)*	*49 (48)*	*1 (1)*			
	*Subclone 3*	*50*	*4 (14)*	*(2)*			*(5)*	*48 (43)*	*2*			
	*Subclone 4*	*50*	*4 (2)*				*1*	*48 (49)*	*1 (1)*			
	*Subclone 5*	*50*	*4*		*2*			*48*				
	*Subclone 6*	*50*	*4*		*1*		*1*	*48*				
	Subclone 7	50	8			1	2	46	1			
	Subclone 8	50	12				1	44	5			
	Subclone 9	50	30			1	5	35	9			
**Wildtype 3**	*Parental*	*50*	*14*		*1*		*3*	*43*	*3*			
	*Subclone 1*	*50*	*6*				*3*	*47*				
	Subclone 2	50	8				3	46			1	
	Subclone 3	50	12	1			1	44	3	1		
	Subclone 4	50	14				4	43	3			
	Subclone 5	50	92					4	46			

Chromosome counts on wildtype iPSC subclones. Parental IPSC clone ID#s correspond to clones listed in Table 2. The subclones that had at least two fold reduced aneuploidy rates compared to their parental iPSC clones are in italic typeface. Subclone 2-1, 2-2, 2-3, and 2-4 were re-examined after 19 days in culture and the karyotype is shown in parenthesis.

## Discussion

**Figure 6:**
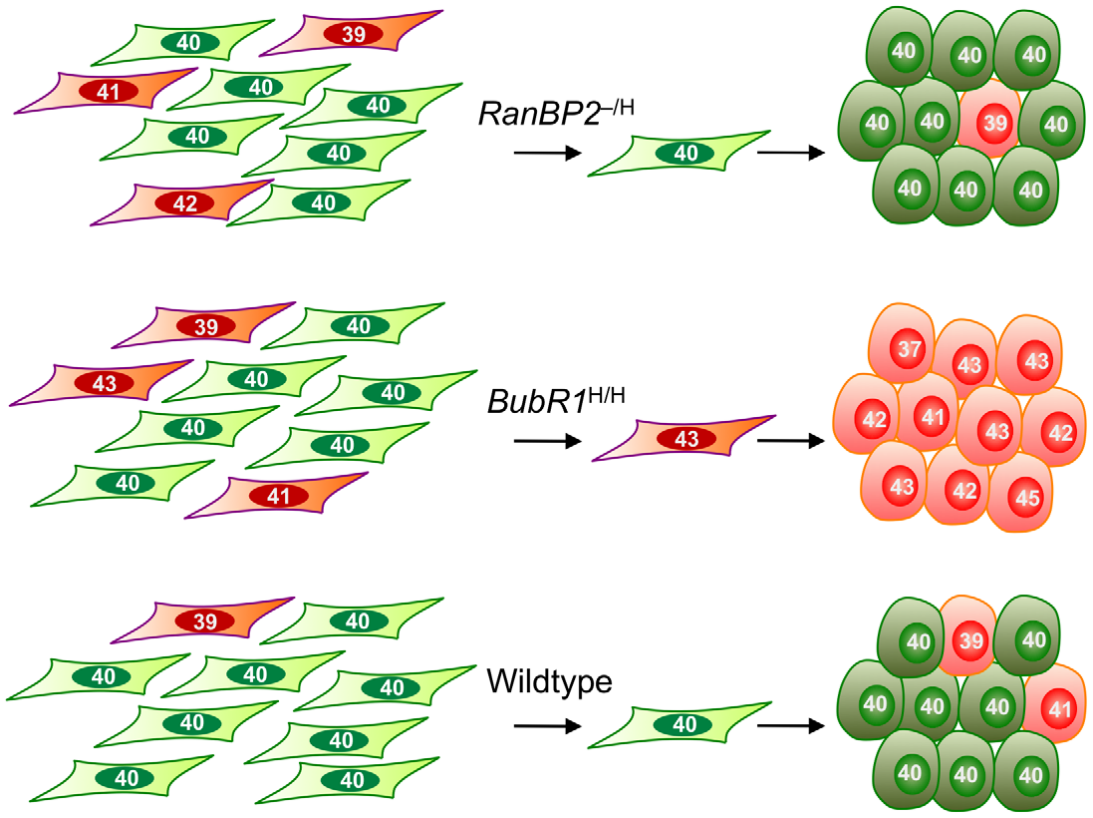
Model illustrating the contrasting effects of W-CIN gene defects on iPSC genomic integrity. *RanBP2* hypomorphic iPSCs originate from MEFs with normal chromosome numbers and exhibit a high degree of chromosome number stability. *BubR1* hypomorphism results in selective reprogramming of aneuploid cells and yields chromosomally unstable iPSCs. iPSC cultures established from wildtype MEFs typically contain relatively small subpopulations of cells with abnormal chromosome numbers.

Although the importance of iPSC technology for regenerative therapies is broadly recognized, several hurdles to their clinical use exist, including the potential of genomic instability. Here we have examined the relationship between somatic cell reprogramming and W-CIN, a type of genomic instability associated with cancer and other human disorders. Our studies provide several important new insights that should improve the efficacy of iPSC use in future clinical applications. First, we demonstrate that W-CIN does not pose a barrier to reprogramming. Second, we show that W-CIN iPSCs are capable of differentiating into all three distinct germ layer cell types. Third, we show that although MEFs with two distinct W-CIN defects efficiently reprogram into iPSCs, they do so with highly contrasting outcomes on chromosome number integrity and stability (Figure 6): our data suggest that BubR1 hypomorphic iPSC clones preferentially originate from aneuploid MEFs, while RanBP2 hypomorphic iPSC clones preferentially stem from MEFs with normal diploid chromosome numbers. Fourth, our data uncovered the fascinating concept that a W-CIN gene defect responsible for severe aneuploidization in somatic cells can become dormant upon reprogramming. The particular W-CIN defect that revealed this concept is RanBP2 hypomorphism. We ruled out that a failure to maintain the RanBP2 hypomorphic status after reprogramming is responsible for restoring high-fidelity chromosome segregation. One possibility is that SUMO E3 ligases other than RanBP2, such as PIAS proteins, redundantly targeting Top2a to inner centromeric regions of duplicated chromosomes [40]–[42]. An alternative explanation might be that Top2a accumulates to inner centromeres in a SUMO independent fashion in pluripotent cells. Regardless of the precise mechanism by which W-CIN can be concealed in iPSCs, the phenomenon itself highlights that it will not only be important to check iPSCs for aneuploidization but also the somatic cells from which they originated.

The observation that aneuploid cells within BubR1 hypomorphic MEF cultures undergo preferential reprogramming is puzzling given that BubR1 insufficiency engages the p16^Ink4a^-Rb and p53-p19^Arf^ pathways [43], both of which have been shown to inhibit reprogramming [7], [28]. Perhaps activation of these tumor suppressor pathways is necessary but not sufficient for the elimination of aneuploid MEFs during the early stages of reprogramming. The observation that *BubR1* ^H/H^ aneuploid MEFs preferentially dedifferentiate raises the possibility that BubR1 might be a key component of a surveillance pathway that prevents aneuploid cells from reprogramming. Interestingly, in earlier studies we have shown that BubR1 levels decrease with aging in various mouse tissues [22], [44], [45]. This, together with the observation that aneuploid MEFs with low amounts of BubR1 readily reprogram into chromosomally unstable iPSCs implies that reprogramming of somatic cells from elderly individuals into karyotypically normal and stable iPSCs may be particularly challenging. It will be interesting to further explore this possibility by testing whether restoration of high BubR1 levels in somatic cells of older individuals would improve iPSC quality.

Mitosis is more prone to errors when cells divide in culture as evidenced by the low rates of chromosome missegregation observed in early passage MEFs from wildtype mice [26], [46]. Although the actual cause of such aneuploidies is unknown, it is generally believed that they are induced by cell culture stress [47]. Karyotypically normal mouse ESC lines used in gene targeting experiments are known to acquire severe aneuploidy upon extensive *in vitro* passaging, indicating that pluripotent cells are also susceptible to cell culture-induced chromosome segregation errors [48]. Our finding that aneuploid cells emerge in iPSC cultures originating from karyotypically normal wildtype MEFs (Table 1) confirms earlier indications that reprogrammed cell lines, like ESC lines, are subject to cell culture induced aneuploidization [49]. Since aneuploidy poses a risk for negative side effects in therapeutic applications, it will be important to devise strategies to avoid it. We find that aneuploidization rates of iPSC clones derived from wildtype MEF cultures can be markedly reduced through subcloning, implying that iPSC cultures contain subsets of cells that are quite resistant to cell culture induced mitotic stress. Thus subcloning might be a pragmatic method to produce iPSC lines with high chromosome integrity.

## Materials and Methods

### Cell Culture

*BubR1* ^H/H^ and *RanBP2* ^–/H^ MEFs were previously established [22], [24]. These MEF lines had a C57BL/6×129Sv/E mixed genetic background. MEFs were grown in DMEM containing 10% FCS, 2 mM L-glutamine, 1 mM sodium pyruvate, 100 µM non-essential amino acids, 55 µg/ml ß-mercaptoethanol, and 10 µg/ml gentamycin. IPSCs were generated and routinely cultured in ES cell medium. This medium consisted of high-glucose DMEM supplemented with 15% FCS, 2 mM L-glutamine, 1 mM sodium pyruvate, 100 µM non-essential amino acids, 55 µg/ml ß-mercaptoethanol, 10 µg/ml gentamycin and 500 U/ml ESGRO LIF (Millipore). SNL cells were obtained from Dr. Allen Bradley [50], [51]. These cells were mitotically inactivated by irradiation (3000 rads) and seeded on plates coated with 0.1% gelatin in PBS. ESCs used in this study were TL1 cells obtained from Dr. Bridgid Hogan. These cells were derived from a 129/Sv mouse blastocyst [52].

### Generation of iPSCs

IPSCs were generated essentially as described in detail elsewhere [53]. Briefly, using the pMXs-IP vectors (obtained via Addgene), retroviruses expressing Oct3/4, Sox2 and Klf4 were produced from Plat-E cells (Cell Biolabs) using Lipofectamine 2000 (Invitrogen). Supernatants were collected 48 h after transfection, passed through 0.45 µm cellulose filter, and mixed 1∶1∶1 (v/v). Eight×10^5^ fibroblasts were seeded onto 10-cm culture plates and the next day infected with 10 ml of viral cocktail in the presence of 4 µg/ml polybrene. Seventy-two h post-infection, the medium was replaced with mouse ES cell medium and refreshed every 1 to 2 days. Colonies were picked 16–26 days post-transduction, trypsinized and seeded in 96 wells coated with irradiated SNL cells. To compare the efficiency of iPSC generation between wildtype and mutant MEFs, 3 lines of wildtype, *RanBP2^–/H^* and *BubR1^H/H^* MEFs were transduced with the same virus cocktail as described above. The number of ES cell-like colonies was manually counted after 3 weeks. To subclone iPSC clones, single cell suspensions were prepared. Two hundred fifty, 500 and 1,000 iPSCs were seeded on 10-cm dishes with SNL feeders. IPSC colonies were picked and clonally expanded.

### Growth Rate Analyses

Growth curves of MEFs were generated using 3 independent MEFs lines for each of the indicated genotypes. P4 MEFs were recovered from frozen stocks. The next day (day 0), 1.5×10^4^ MEFs were seeded into 35-mm dishes (in duplicate). Cell counts were performed 24 h after seeding and at 24-h intervals thereafter, for up to 4 days. The average number of cells per each time point was calculated by averaging the average of the duplicates for each of the 3 independent MEF lines. Log cell numbers were calculated by dividing the average number of cells counted on each of the days by the number of cells seeded. To determine the growth rates of iPSCs, 3 wildtype, 3 *BubR1* ^H/H^ and 3 *RanBP2* ^–/H^ iPSC clones were seeded in duplicate at 1×10^5^ cells per well of a 6-well plate density. Every 3 days, we trypsinized the cultures, counted the number of cells, and reseeded 1×10^5^ cells. This process was repeated 9 times. For each iPSC line, the average between duplicates was calculated. The growth curves for each genotype were plotted as the average of the three corresponding cell lines. The cumulative cell numbers are indicated on the Y-axis on a logarithmic scale.

### Testing for Complete Reprogramming to Pluripotency

#### Retroviral silencing

iPSCs were grown in the presence of 4 µg/ml puromycin. After 24 h, cells were examined for viability using an inverted microscope.

#### ES markers (RT–PCR)

For ES cell and iPSC clones, feeder cells were removed from culture by incubating the mixed cell suspension in gelatin-coated dishes for 30 min. RNA was isolated from cells using Trizol (Invitrogen). Reverse transcription was performed using Superscript III and the random hexamer primer (Invitrogen). PCR was performed using Platinum taq (Invitrogen). The forward primers for exogenous Oct4, Sox2 and Klf4 and the primer sets for endogenous Oct4, Sox2, Klf4, Nanog, Rex1, Esg1 and G3pdh were described previously [54]. The reverse primer for exogenous Oct4, Sox2, and Klf4 was 5′-ATATCAAGCTTATCGAGCGGC-3′.

#### Differentiation markers (qRT–PCR)

mESCs or iPSC clones were differentiated by culturing in suspension at 2×10^6^ cells/ml in ES medium without LIF. EBs were collected after 5 and 10 days for RNA isolation. Preparation of cDNA was done as above. Real-time PCR was performed using SYBR green master mix (Invitrogen) with 95°C for 5 min, 40 cycles at 95°C for 15 sec, 60°C for 30 sec, 72°C for 30 sec, followed by a dissociation cycle. Fold changes in gene expression in EBs (day 5 and day 10) vs. iPSCs (day 0) were calculated based on the 2^−ΔΔCT^ method and normalized to GAPDH. The primers used were described previously [32].

#### Teratoma formation

iPSCs growing on SNL feeders in one well of a 6-well plate were trypsinized, washed once with ES medium, and suspended in ES medium at 4×10^6^ cells per ml. 2×10^6^ cells were injected into the subcutaneous tissue above the rear haunch of 5 to 8 week old C.B-17 SCID males (Taconic #CB17SC-M) using a 23GX1 needle. Twenty one days post injection, teratomas were dissected, photographed, measured using a digital caliper (World Precision Instruments, Inc. #501601), fixed in 10% formalin for 20 h and processed for paraffin embedding. Sections were prepared and routinely stained with hematoxylin and eosin and scored for the presence of tissues derived from all three germ layers as previously described [8]. As controls we used mESCs (TL1), wildtype and *BubR1^H/H^* MEFs, and ES medium alone. The tumor volume values were plotted using Prism 4.0a for Mac. Pictures were acquired using an Olympus AX70 microscope with Olympus DP71 color camera, UPlanFl 20×/0.50 Olympus objective and DP Controller 3.1.1.267 software.

#### Chimera formation

Wildtype, *BubR1* ^H/H^ and *RanBP2* ^–/H^ iPSC clones growing on SNL feeders were trypsinized and injected into BALB/c host blastocysts (Harlan) using standard procedures.

### In Vitro Differentiation of iPSCs

Wildtype and *RanBP2^–/H^* iPSC cultures were trypsinized, collected in 8 ml ES cell medium and plated onto a gelatin-coated 10-cm dish for 1 h to allow SNL feeders to attach. The supernatant was then collected, pelleted and resuspended in ES cell medium without LIF. 2×10^6^ cells in 10 ml medium were transferred to a Petri dish to induce EB formation. The medium was changed every 2 days by collecting the suspension in 15 ml falcon tube, leaving the cells to settle at the bottom, then replacing the supernatant with fresh medium. After 8 days, EBs were collected and seeded onto gelatin-coated 10-cm culture dish for outgrowth of differentiated cells. Karyotyping was performed 5 days thereafter.

### Indirect Immunofluorescence and Western Blotting

For immunofluorescence, iPSCs were seeded on 0.1% gelatin-coated glass slides. After 24–48 h, the cells were fixed in 4% paraformaldehyde for 10 min at room temperature, washed 3× with PBS and permeabilized/blocked for 15 min in PBS containing 0.1% Triton X-100 and 5% FCS. The cells were then incubated with primary antibodies against OCT3/4 (1∶50, mouse monoclonal, sc-5279, Santa Cruz Biotechnology), NANOG (1∶100, rabbit polyclonal, A300-398A, Bethyl Laboratories Inc.), and SSEA1 (1∶100, mouse monoclonal, Developmental Studies Hybridoma Bank) for 1 h in PBS containing 0.1% Triton X-100 and 5% FCS. The cells were washed 3× with PBS and incubated with the appropriate AlexaFluor-conjugated secondary antibodies (Invitrogen). After Hoechst staining, the cells were mounted in Vectashield mounting medium (Vector Labs). For Top2a staining, the cells were treated with 0.1 µM monastrol (Biomol, GR322-0025) for 3 h before fixation and the staining was performed as previously described [24]. Western blotting for RanBP2 and BubR1 was performed as previously described [24], [55]. Antibodies from TopoGEN (#2010-1A) and Santa Cruz (SC-6243) were used to detect Top2a and p53, respectively. The antibody against p16 was as previously described [25], [27].

### Chromosome Counting

iPSC clones (or subclones) growing on SNL feeders were incubated for 4 h with 0.8 µg/ml KaryoMax Colcemid (Invitrogen). The cells were then harvested, incubated in 0.075 M KCl solution for 12 min at 37°C and fixed in 3∶1 methanol to acetic acid (v/v) solution. After two washes with fixative, the iPSC suspension was dropped onto glass slides and dried on wet paper towels. The slides were stained with KaryoMAX Giemsa staining solution (Invitrogen) according to the manufacture's instructions. Chromosome numbers of 50 or 100 metaphase spreads were counted for each iPSC line.

## Supporting Information

Figure S1 Analysis of cell cycle inhibition before and after reprogramming. (A) Growth curves of P5 MEFs. Curves were generated from three independent clones per genotype seeded in duplicates. Error bars represent SD. (B) Western blot analysis of wildtype and *BubR1* ^H/H^ iPSC extracts probed for p53 and p16. Actin served as a loading control.
(TIF)

Figure S2 Potential models for selective reprogramming of aneuploid *BubR1* ^H/H^ MEFs. We observed that >90% *BubR1* ^H/H^ iPSC clones have a majority population consisting of a chromosome number other than 40 even though only 38% of MEFs were aneuploid at the onset of reprogramming. Two possible mechanisms, designated A and B, might explain this observation. According to mechanism A (highlighted in black font), the chromosome number of the founding MEF cell at the onset of reprogramming represents the chromosome number of the majority population of the iPSC clone. This mechanism would indicate a bias for reprogramming of karyotypically abnormal *BubR1* ^H/H^ MEF cells. According to mechanism B (highlighted in red font), the chromosome number of the founding MEF cell does not represent the chromosome number of the majority population of the iPSC clone due to a sharp increase in aneuploidization rates when cells reach the reprogrammed state. This mechanism would even be consistent with a bias against reprogramming of karyotypically abnormal MEF cells. If mechanism B holds true, one would expect to see no correlation between the spectrum of chromosome losses and gains of a parental iPSC clone and its single cell-derived subclones.
(TIF)

Figure S3 Measurement of the degree of RanBP2 insufficiency in *RanBP2* ^–/H^ iPSC clones. (A) Western blot analysis of serially diluted *RanBP2^+/+^* iPSC cell lysates for RanBP2 and actin. (B) The average RanBP2 signal intensity of 3 independent *RanBP2^+/+^* iPSC clones plotted against percentage of lysate volume loaded using the indicated equation. (C) Relative RanBP2 protein amount in *RanBP2* ^–/H^ iPSC clones. Lysates from the indicated *RanBP2* ^–/H^ iPSCs clones and from wildtype iPSC clones were subjected to western blot against RanBP2 and actin. Using Image J, the RanBP2 signal was calculated, normalized to background, normalized to actin, and then averaged between duplicates. The value for each *RanBP2* ^–/H^ iPSCs clone was then normalized to wildtype. The relative RanBP2 protein amount (%) was then calculated with the graph and equation in (B).
(TIF)
