# Supplementary figures and images for: Reprogramming to Pluripotency Can Conceal Somatic Cell Chromosomal Instability
Source: PLoS Genet. 2012 Aug 30;8(8):e1002913. doi: 10.1371/journal.pgen.1002913 (PMC3431347; doi:10.1371/journal.pgen.1002913)

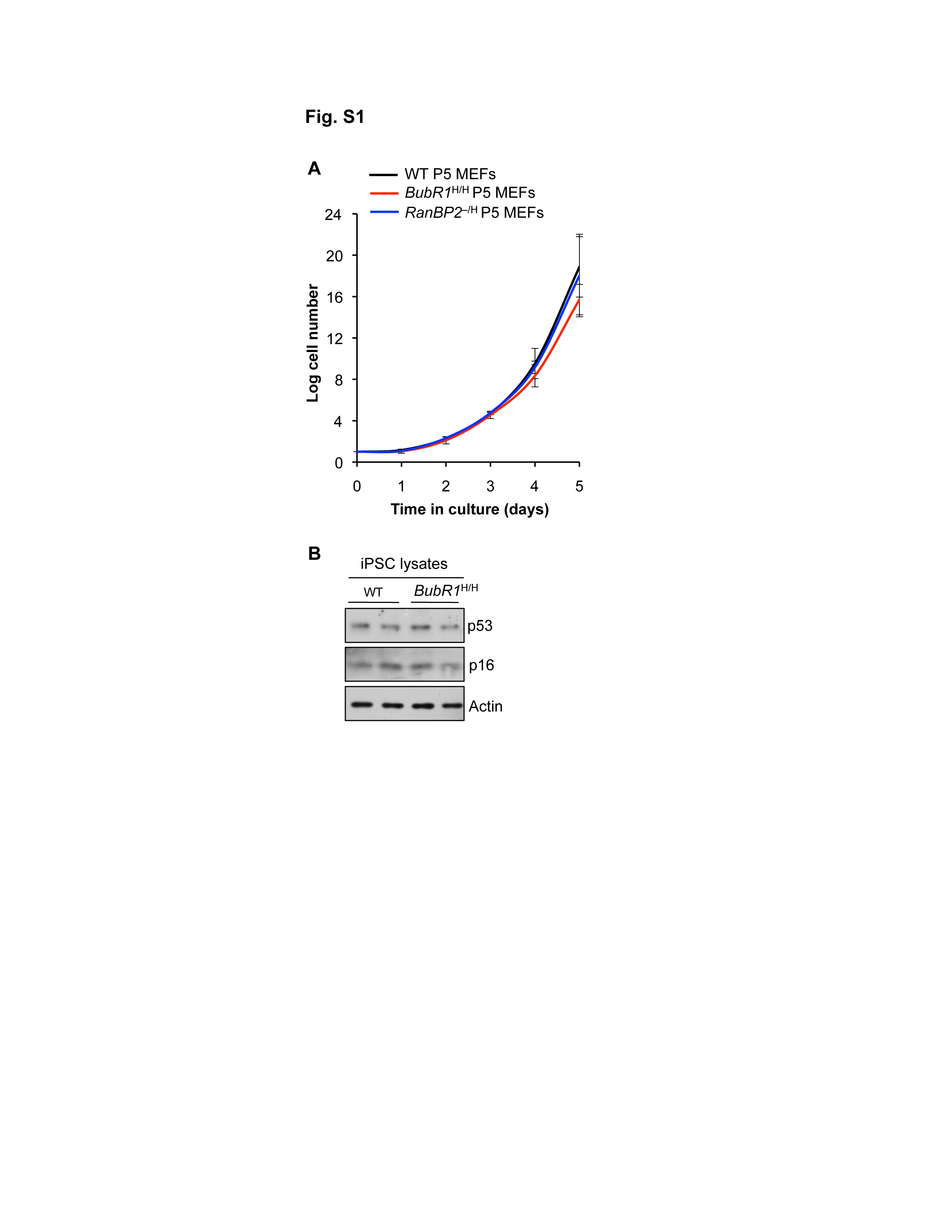

Supplement: Figure S1 — Analysis of cell cycle inhibition before and after reprogramming. (A) Growth curves of P5 MEFs. Curves were generated from three independent clones per genotype seeded in duplicates. Error bars represent SD. (B) Western blot analysis of wildtype and BubR1 H/H iPSC extracts probed for p53 and p16. Actin served as a loading control. (TIF) [file pgen.1002913.s001.tif]

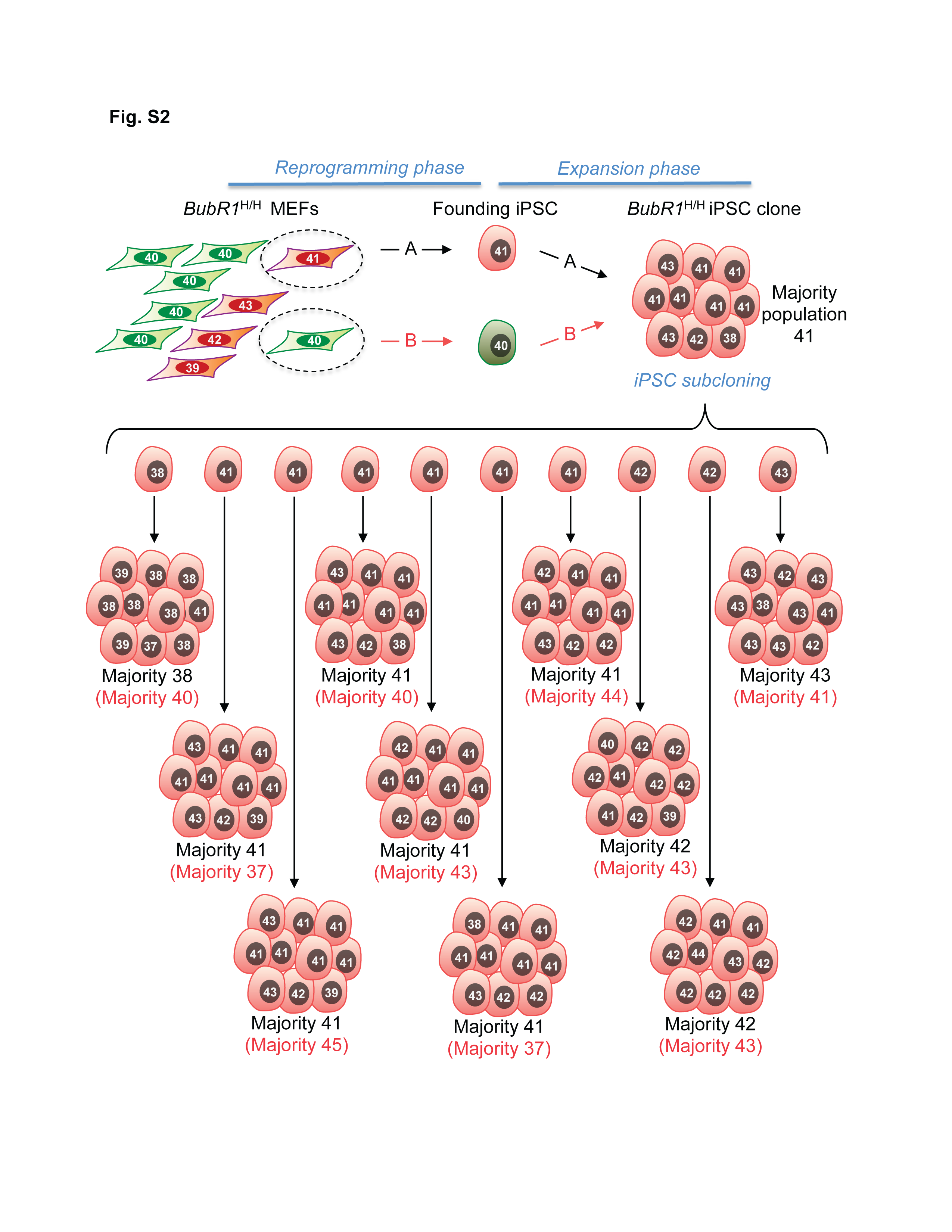

Supplement: Figure S2 — Potential models for selective reprogramming of aneuploid BubR1 H/H MEFs. We observed that >90% BubR1 H/H iPSC clones have a majority population consisting of a chromosome number other than 40 even though only 38% of MEFs were aneuploid at the onset of reprogramming. Two possible mechanisms, designated A and B, might explain this observation. According to mechanism A (highlighted in black font), the chromosome number of the founding MEF cell at the onset of reprogramming represents the chromosome number of the majority population of the iPSC clone. This mechanism would indicate a bias for reprogramming of karyotypically abnormal BubR1 H/H MEF cells. According to mechanism B (highlighted in red font), the chromosome number of the founding MEF cell does not represent the chromosome number of the majority population of the iPSC clone due to a sharp increase in aneuploidization rates when cells reach the reprogrammed state. This mechanism would even be consistent with a bias against reprogramming of karyotypically abnormal MEF cells. If mechanism B holds true, one would expect to see no correlation between the spectrum of chromosome losses and gains of a parental iPSC clone and its single cell-derived subclones. (TIF) [file pgen.1002913.s002.tif]

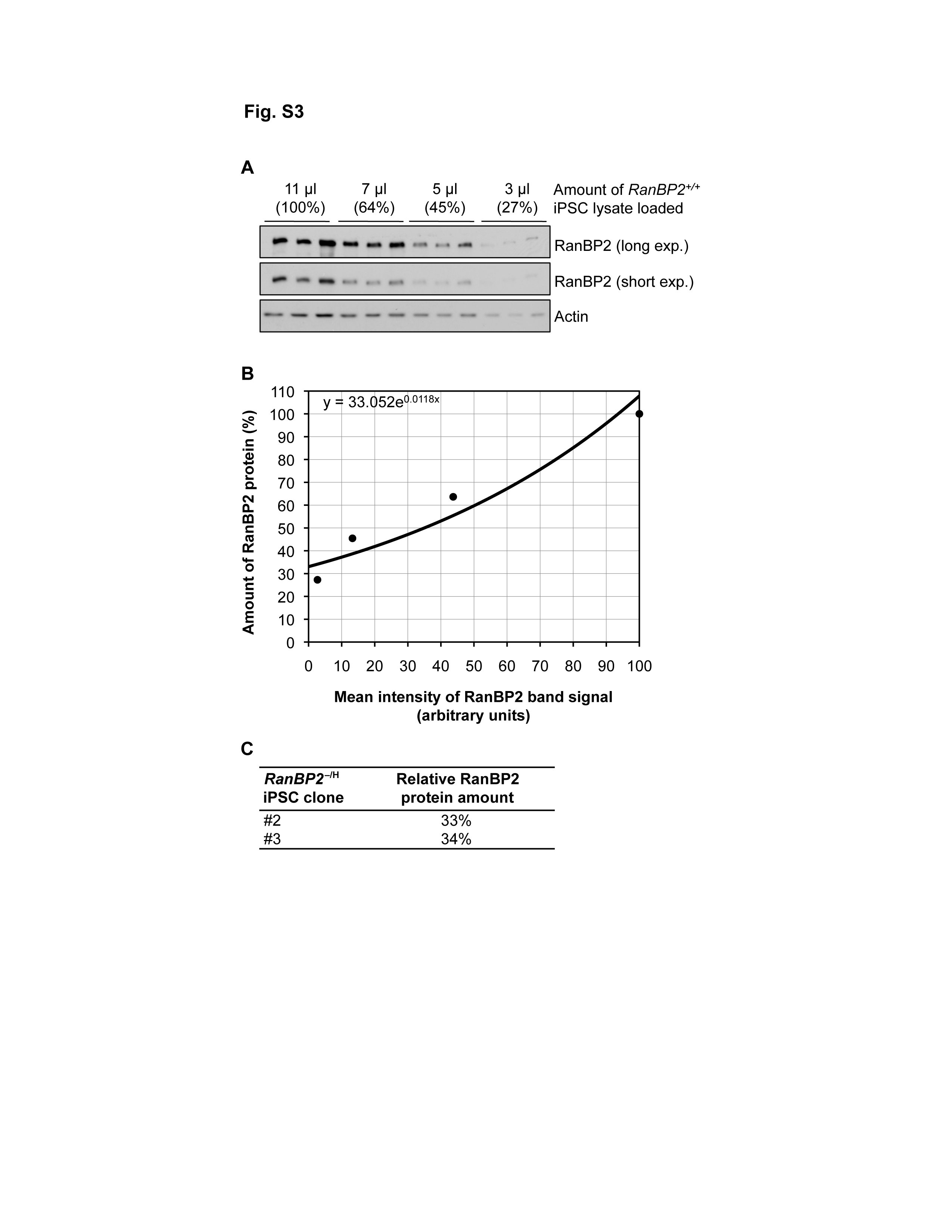

Supplement: Figure S3 — Measurement of the degree of RanBP2 insufficiency in RanBP2 –/H iPSC clones. (A) Western blot analysis of serially diluted RanBP2+/+ iPSC cell lysates for RanBP2 and actin. (B) The average RanBP2 signal intensity of 3 independent RanBP2+/+ iPSC clones plotted against percentage of lysate volume loaded using the indicated equation. (C) Relative RanBP2 protein amount in RanBP2 –/H iPSC clones. Lysates from the indicated RanBP2 –/H iPSCs clones and from wildtype iPSC clones were subjected to western blot against RanBP2 and actin. Using Image J, the RanBP2 signal was calculated, normalized to background, normalized to actin, and then averaged between duplicates. The value for each RanBP2 –/H iPSCs clone was then normalized to wildtype. The relative RanBP2 protein amount (%) was then calculated with the graph and equation in (B). (TIF) [file pgen.1002913.s003.tif]
